# Congenic Mice Provide Evidence for a Genetic Locus That Modulates Spontaneous Arthritis Caused by Deficiency of IL-1RA

**DOI:** 10.1371/journal.pone.0068158

**Published:** 2013-06-28

**Authors:** Yanhong Cao, Xiaoyun Liu, Nan Deng, Yan Jiao, Yonghui Ma, Karen A. Hasty, John M. Stuart, Weikuan Gu

**Affiliations:** 1 Institute of Kaschin-beck Disease, Center for Endemic Disease Control, Chinese Center for Disease Control and Prevention, Harbin Medical University; Key Laboratory of Etiologic Epidemiology, Education Bureau of Heilongjiang Province and Ministry of Health (23618104), Harbin, China; 2 Departments of Orthopaedic Surgery and Biomedical Engineering, University of Tennessee Health Science Center, Memphis, Tennessee, United States of America; 3 Department of Medicine, University of Tennessee Health Science Center, Memphis, Tennessee, United States of America; 4 Research Service, Veterans Affairs Medical Center, 1030 Jefferson Avenue, Memphis Tennessee, United States of America; INSERM-Université Paris-Sud, France

## Abstract

To understand the role of genetic factors involved in the development of spontaneous arthritis in mice deficient in IL-1 receptor antagonist protein (IL_1RA), we have identified a genomic region containing a major quantitative trait locus (QTL) for this disease. The QTL is on chromosome 1 and appears to be the strongest genetic region regulating arthritis. To confirm the importance of the QTL and to identify potential candidate genes within it, we conducted speed congenic breeding to transfer the QTL region from DBA/1 mice that are resistant to spontaneous arthritis into BALB/c^−/−^ which are susceptible. Genetic markers along every chromosome were used to assist in the selection of progeny in each generation to backcross to BALB/c^−/−^. By the 6th generation we determined that all of the chromosomes in the progeny were of BALB/c origin with the exception of portions of chromosome 1. At this stage we intercrossed selected mice to produce homozygous strains containing the genomic background of BALB/c^−/−^ except for the QTL region on chromosome 1, which was from DBA/1. We were able to establish two congenic strains with overlapping DBA/1 DNA segments. These strains were observed for the development of spontaneous arthritis. Both congenic strains were relatively resistant to spontaneous arthritis and had delayed onset and reduced severity of disease. The gene/s that regulates this major QTL would appear to be located in the region of the QTL that is shared by both strains. The common transferred region is between D1Mit110 and D1Mit209 on chromosome 1. We evaluated this region for candidate genes and have identified a limited number of candidates. Confirmation of the identity and precise role of the candidates will require additional study.

## Introduction

Interleukin 1 (IL-1) is a major contributor to the development of immune mediated arthritis. This cytokine is expressed by macrophages, monocytes and synovial fibroblasts. Its action is in part regulated by the IL-1 receptor antagonist protein (IL-1RA) which is the product of the Il1rn gene. The importance of IL-1RA in regulation of IL-1 activity has been established by generating mice deficient in IL-1RA. These IL-1RA deficient mice develop spontaneous arthritis as first described by Horai and colleagues [1]. BALB/c mice that are homozygous for the deficiency (BALB/c^−/−^) develop inflammation in the hind limbs beginning at about 6 weeks of age and achieving an incidence approaching 100% by 3 months of age. Histopathologic examination of the joints of these mice shows infiltration of inflammatory cells and synovial proliferation. The development of disease is in part dependent upon genetic background since DBA/1 mice with a similar deficiency in IL-1RA do not develop spontaneous arthritis [2].

Deficiency of IL-1RA (DIRA) as a result of IL1RN mutation has also been identified in humans and results in a rare autosomal recessive autoinflammatory syndrome. DIRA is manifested by systemic inflammation including rash, painful movement, joint swelling and bone involvement [3]. Polymorphism of this gene in humans has also been associated with increased risk of osteoporotic fractures [4] and with gastric cancer [5]. Although the manifestations of IL-1RA deficiency in humans are somewhat different from those observed in mice, it is clear that IL-1RA is involved in human arthritis. Recombinant human IL-1RA has been developed as the therapeutic product Anakinra [6]. Administration of Anakinra has been shown to alleviate rheumatoid arthritis [7] and several other inflammatory disorders including systemic-onset juvenile idiopathic arthritis, familial Mediterranean fever and others. Because of its involvement in human disease there has been substantial interest in the mechanisms by which IL-1RA modulates arthritis.

Spontaneous arthritis is dependent not only on IL-1RA deficiency but also other as yet unidentified genetic factors. In order to identify those factors we used classical genetic techniques and bred susceptible and resistant mice to obtain an F2 generation and identified QTL associated with arthritis susceptibility [8]. After we conducted QTL analysis with phenotypic and genotypic determination of 191 F2 progeny, we obtained evidence for potential QTL on chromosomes 1, 6, 11, 12, and 14 [8]. The data suggested that the QTL on chromosomes 1 and 6 had the greatest influence on disease whereas there was weaker evidence for the involvement of potential QTL on chromosomes 11, 12, and 14 [8]. The QTL on chromosome 1 covers a large region at the distal end of the chromosome. Because of the strength of the association of spontaneous arthritis and this QTL we undertook additional studies to further characterize it. We hypothesized that one gene or a few genes within the QTL region regulate spontaneous arthritis. Accordingly, if a genomic fragment that contains the gene/s responsible for regulation of IL-1RA and development of spontaneous arthritis in BALB/c was replaced by the analogous fragment from DBA/1 mice which are resistant to spontaneous arthritis, then disease in the new strain would be reduced in incidence and/or severity. To test our hypothesis, we have developed congenic strains that contain the genomic fragments in the region of the QTL from DBA/1 mice on a BALB/c background.

Congenic strains are animals in which a genetic locus (often containing a QTL of interest) has been moved from one strain/line (donor) to the background of another strain/line (recipient) by back-crossing. Polymorphism of molecular markers is used to detect the source strain of the genome components of a congenic strain. For these experiments, we used speed or marker-assisted congenic breeding. Theoretically, the classical protocol of congenic breeding needs about 10 generations, at which 99+% of the genetic background of the progeny is of recipient origin while still retaining heterozygosity at the region of interest [9]–[10]. However, the availability of dense genetic maps of the mouse genome has allowed the development of marker-assisted breeding strategies [10], which reduce the number of generations required to eliminate donor strain-derived alleles outside the genetic region of interest. By employing this strategy of “speed congenics” we were able to rapidly produce the mouse strains used in this study.

## Materials and Methods

### Mice

All mice have been maintained in the animal facility of the Department of ‘Veterans Affairs Medical Center, Memphis. Experimental animal procedures and mouse husbandry were performed in accordance with the National Institutes of Health’s Guide for the Care and Use of Laboratory Animals and approved by the VAMC Institutional Animal Care and Use Committee.

### Microsatellite Markers

A total of 123 microsatellite markers were selected for genotyping of progeny to assist with identifying the most informative backcrosses for breeding (Table S1). Those 123 markers are polymorphic between the two parental strains DBA/1 and BALB/c, and are evenly distributed along the 19 autosomal chromosomes, with distances of less than 20 cM from each other. The number of markers on each of chromosomes is 19, 8, 9, 6, 6, 6, 6, 6, 6, 7, 5, 4, 8, 5, 5, 4, 3, 5, 5 from chromosome 1 to 19, respectively.

### Genotyping

Genomic DNA was extracted from tissues obtained by ear punch. The procedure used has been previously described [8]. Briefly, DNA was extracted from the tissue and amplification of microsatellite markers conducted by polymerase chain reaction (PCR). PCR products were analyzed using poly-acrylamide gel electrophoresis using the Mega-Gel Dual High-Throughput Vertical Electrophoresis System (C.B.S. Scientific, Del Mar, CA).

### Breeding Procedure

The following procedure was used for the congenic breeding (Figure S1): 1) Mice with IL-1RA deficiency on the BALB/c background were crossed with DBA/1 mice which were also deficient in IL-1RA to produce heterozygous (F1) mice; 2) The F1 progeny were backcrossed to BALB/c^−/−^ to produce N1 mice. The N1 mice were genotyped with 123 microsatellite markers. The individual mice with the fewest genomic markers for DBA/1 background but with the QTL region from the DBA/1 on chromosome 1 were selected for the next generation; 3) The selected N1 mice back cross to BALB/c^−/−^ to produce an N2 generation. The N2 mice were then genotyped for the same 123 microsatellite markers. The individual mice with the most BALB/c^−/−^ genetic background but possessing DBA/1 genomic DNA within the heterozygous QTL region on chromosome 1 were selected for the next generation; 4) The selected N2 mice were backcrossed to BALB/c^−/−^ to produce the N3 generation. This process was repeated with selection of individual mice with the most BALB/c^−/−^ background as well as the heterozygosity for DBA/1 at the QTL region for six generations; 5) At the end of the sixth generation it was determined that selected mice were homozygous for BALB/c^−/−^ background but contained heterogygous DBA/1 in the region of the QTL; 6) These mice were then interbred to generate BALB/c^−/−^ which were homozygous congenic for DBA/1 within the QTL;7) Ultimately we were able to establish 2 congenic strains. We first obtained the congenic strain BALB.D1-1^−/−^. The transferred region from DBA/1 is from marker D1Mit55 to D1Mit209, which are located at 155166854–155167004 bp and 191493187–191493284 bp, respectively. The second congenic strain is BALB.D1-2^−/−^. The transferred genomic region from DBA/1 is from D1Mit359 to the distal end of chromosome 1; and 8) The new congenic strains were then observed for the development of spontaneous arthritis.

### Phenotype Evaluation

Mice from the BALB/c.D1^−/−^ congenic strain were observed for the development of spontaneous arthritis. Individual mice were visually inspected for the presence of arthritis at least three times weekly. Each limb was scored on a scale of 0–4. Statistics used for the analysis of arthritis severity/incidence were as same as we have previously described ((0-no evidence of erythema and swelling, 1- mild redness and swelling of joint and ankle, 2-definite swelling, 3-severe swelling of entire limb, and 4-limb burned out and deformed). A severity score was calculated for the 4 limbs. The maximum score for an individual mouse is 16 [2], [8]. The disease severity of a mouse is calculated with total scores of all limbs divided by the maximal score possible (in this case, 16) and multiplied by 100. The disease severity (total score of arthritis) of a strain is calculated with total scores of all mice divided by the total number of mice. The incidence was calculated with the number of affected mice divided by the total number of mice [2], [8]. Differences were analyzed by Fisher’s exact test for incidence and by ANOVA for severity.

### Cytokine Assays

Cells were harvested from mouse spleens at 4 months of age. The spleens were dissociated, washed and cultured in HL-1 medium. Cells were cultured in 48 well plates at a density of 2 × 10^6^ per well and CD3CD28 stimulation beads (Life Technology) added at the initiation of culture. Supernatant fluids were removed after 48 hours and cytokine levels were measured using Milliplex kits by Multianalyte Technology (Millipore, MA) according to the manufacturer’s protocol. Procedures for assay sensitivity, precision and accuracy were based on the overnight protocol. Briefly, minimum detectable concentration is calculated by the StatLIA® immunoassay analysis software from Brendan Technologies (Carlsbad, CA). Intra-assay precision is generated from the mean of the %CV’s from 8 reportable results across two different concentration of cytokines in one experiment. Inter-assay precision is generated from the mean of the %CV’s from 4 – 8 reportable results across two different concentrations of cytokine in 4 different experiments. For accuracy, the data represent mean percent recovery of six levels of spiked standards in serum matrices.

### Identification of Candidate Genes

Evaluation of genes within the QTL region of chromosome 1 was conducted using a searching tool, PGMapper (http://www.genediscovery.org/pgmapper/index.jsp) [11]. The procedures used were to that previous published [12]. Briefly, query terms were the combination of the name of the gene with any of these key words: arthritis, inflammation, anti-inflammatory, inflammatory mediator, inflammatory cytokine, autoimmune, immune, joint damage, T-cell, macrophage, neutrophil, angiogenesis, synovial, synovial hyperplasia, synovial fibroblast, lymphocytic infiltrate, and cartilage degradation. For any potential candidates, at least the abstract of one reference was read by two authors to ascertain whether there was a reason to believe that there might be an association between the gene and arthritis. For a gene with more than one reference that indicated its candidacy, at least two references were reviewed to confirm the potential association between the gene and arthritis.

### Bioinformatics Analysis of Candidate Genes

To obtain information on polymorphism and SNPs of every candidate, we searched every gene including at least 2 kb of DNA up and downstream using the Mouse SNP database at the Jackson Laboratory: http://informatics.jax.org/javawi2/servlet/WIFetch?page=snpQF. Input terms for SNP database are two strains, BALB/cj and DBA/1j and the name of each gene in every search. For the information on gene differential expression, we used the list of genes that are differentially expressed in the spleen between BALB/c and DBA/1 (for data in Table S2) in our previous publication [8]. Splenic gene expression patterns in the four arthritis susceptible- (BALB/c-based) and four resistant- (DBA/1-based) IL1ra knockout mice were analyzed using the Affymetrix microarray platform. Statistical analysis for gene expression (expressed as a P value) was determined using EDGE software [8]. To analyze correlation of expression between candidate genes and *Il1rn*, including *Il-1r, Il1a* and *Il1b*, we took full advantage of existing data of gene expression profiles at GeneNetwork at http://www.genenetwork.org/webqtl/main.py. We used gene expression data from spleens obtained from a set of recombinant inbred (RI) strains derived by crossing C57BL/6J (B6) and DBA/2J (D2) and inbreeding progeny for 20 or more generations [13]. Data was generated using the Affymetrix GeneChip Mouse Gene 1.0 ST array (http://www.genenetwork.org/webqtl/main.py?FormID=sharinginfo&GN_AccessionId=283). Correlation was examined between expression levels of probes of each gene and probes from *Il1rn*, including *Il-1r, Il1a*, and *Il1b*.

## Results

### Phenotyping of Congenic Strains

**Figure 1:**
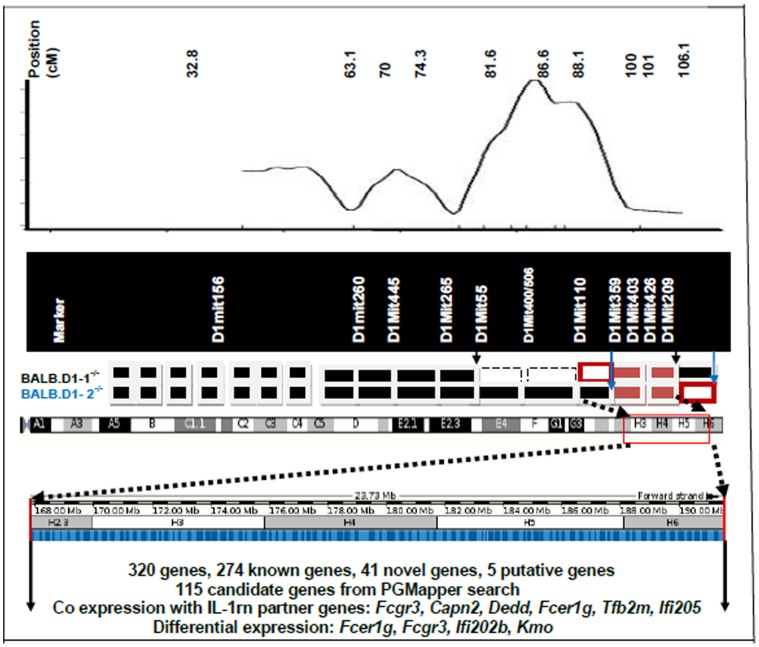
Genomic region of the QTL on chromosome 1 based on genetic markers in two congenic strains. Top is the location of initial mapping of the QTL for arthritis previously identified using the F2 generation. Below that is shown diagrammatically, the transferred genomic regions (white boxes) from DBA/1^−/−^ into Balb/c^−/−^ after the generation of 2 congenic strains. The genomic region from DBA/1 in BALB.D1-1^−/−^ is flanked by two markers, D1Mit55 and D1Mit209. The transferred genomic regions (white boxes) from DBA/1^−/−^ into Balb/c^−/−^ in congenic strain BALB.D1-2^−/−^ is flanked by marker D1Mit359 and the distal end of the chromosome. The minimum overlap region of the transferred genomic regions from DBA/1^−/−^ into Babl/c^−/−^ in congenic strains BALB.D1-1^−/−^ and BALB.D1-2^−/−^ is flanked by markers D1Mit359 and D1Mit209. The most likely QTL genomic region is between D1Mit110 and D1Mit209 with 23.73 Mb which contains 320 genetic elements. Among those genes, 115 are identified as genes relevant to arthritis and its potential pathways. Six genes *Fcgr3, Capn2, Dedd Fcer1g, Tfb2m and Ifi202* are co expressed with IL-1RN and related genes, four genes Fcer1g, Fcgr3, Ifi202b and Kmo are differentially expressed between Balb/c−/− and DBA/1^−/−^.

**Figure 2:**
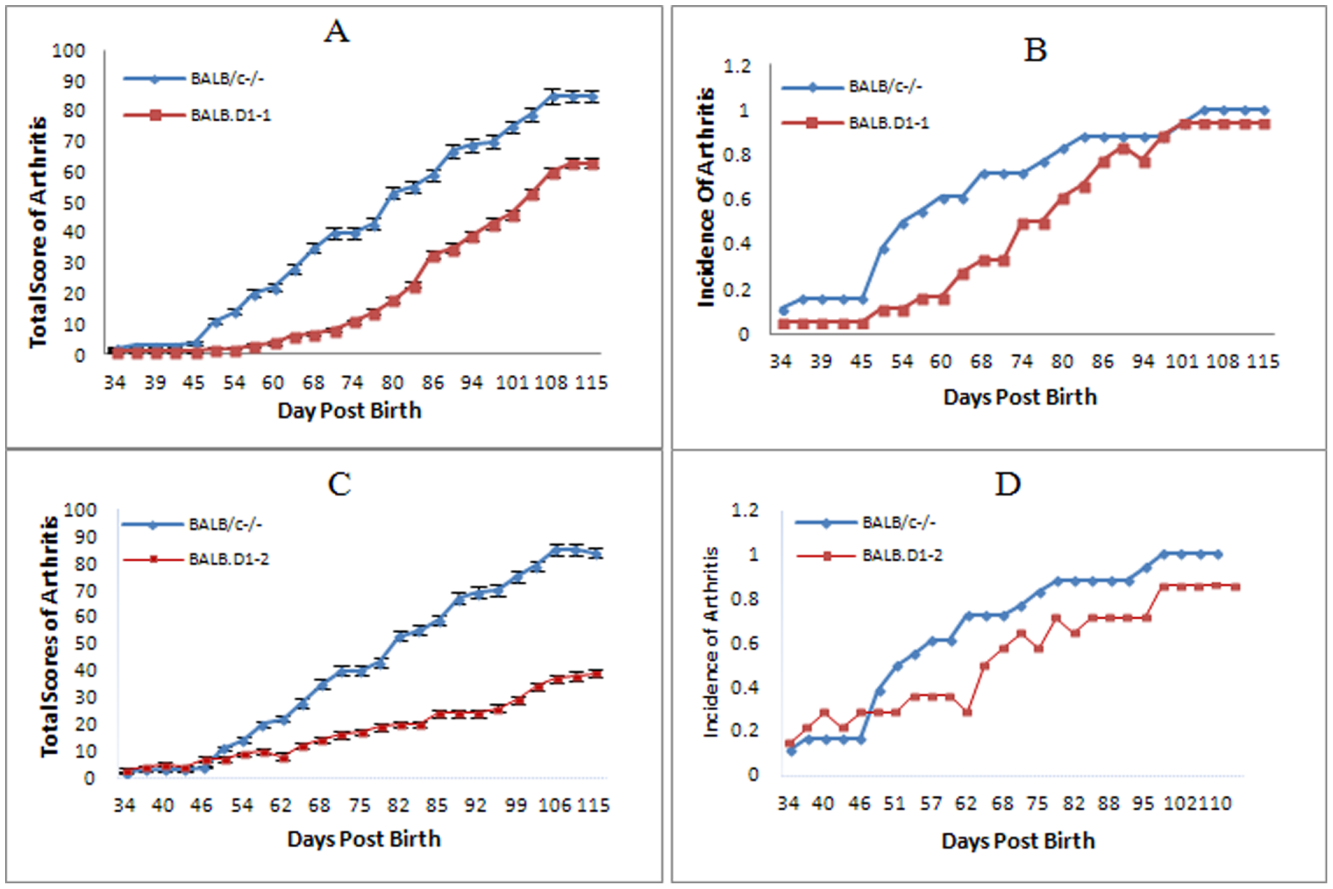
Comparison of arthritis in the parental and congenic strains. The BALB.D1-1^−/−^congenic strain has much less severity (Figure 2A) and delayed onset (Figure 2B) of spontaneous arthritis as compared to the BALB/c^−/−^ parental strain (P< = 0.003 for severity and mean day of onset 74 vs 54 with a P< = 0.05 for incidence). The Phenotype of BALB.D1-2^−/−^ is similar to that of BALB.D1-1^−/−^ even through this strain contains a smaller piece of the genomic region from DBA/1. The phenotype of BALB.D1-2^−/−^ was investigated with 15 mice. In comparison to BALB/c^−/−^, thise congenic strain also had both reduced severity (Figure 2C) and delayed onset (Figure 2D) of arthritis (P value < = 0.007 for severity and mean day of onset 68 vs 54 with P< = 0.01 for incidence). However, the overall incidence of disease in both congenic strains and BALB/c^−/−^ reaches to almost 100% by 105 days of age. For measurement of severity the data are expressed as a percentage of the maximal total score.

**Figure 3:**
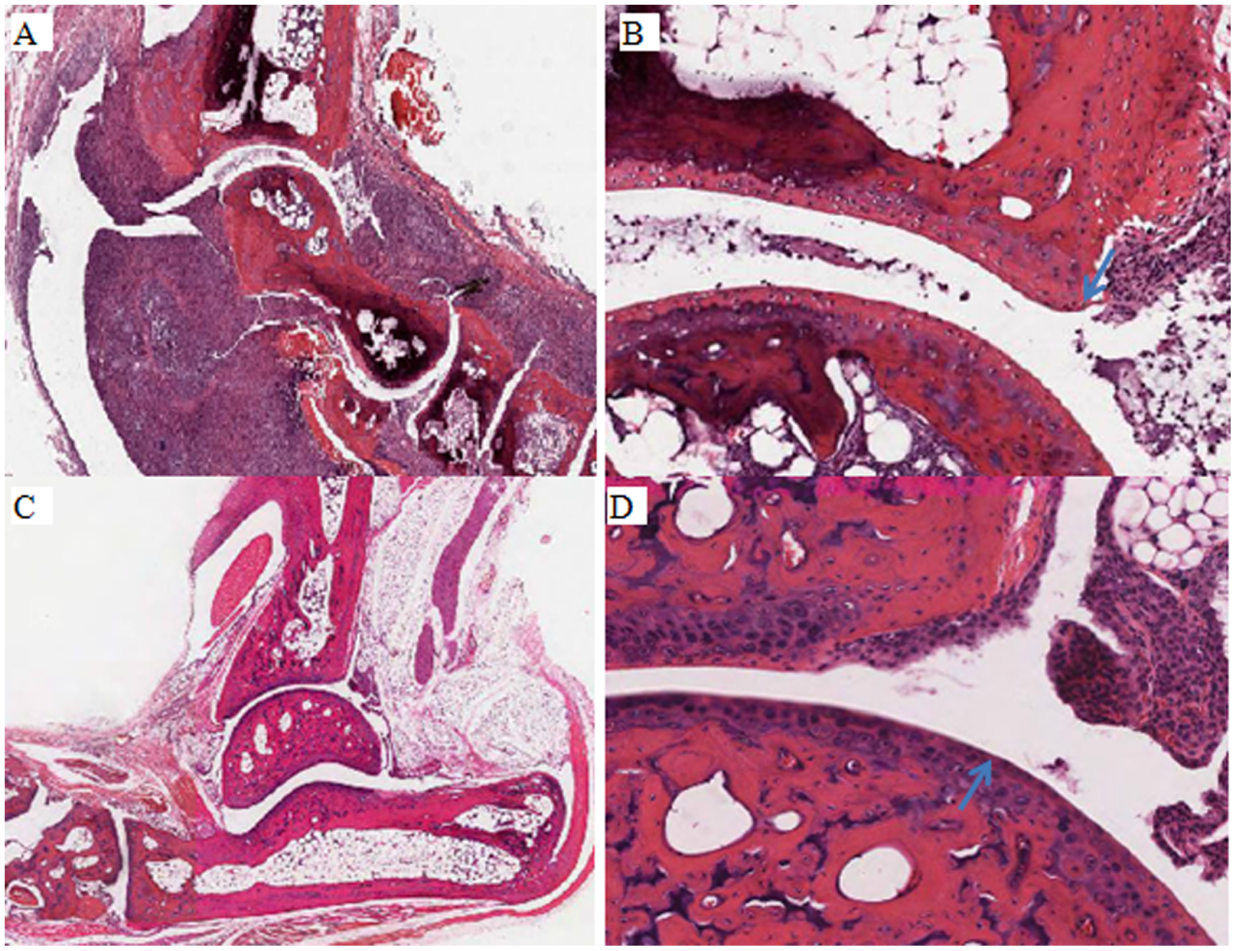
Comparison of arthritis severity in a BALB/c^−/−^ (A, B) and Congenic BALB/c.D1-1^−/−^ mouse (C, D) Panels A and C are cross section of the ankle joint. Panels B and D are higher power views to illustrate the development of an early erosion in the BALB/c^−/−^ mouse whereas the comparable area of the congenic mouse shows only synovitis without erosive disease.

Shown in Figure 1 is the genetic mapping of the QTL on chromosome 1 that was most strongly associated with the development of arthritis in the F2 generation as we have previously described [8]. Strain BALB.D1-1 contains genetic DNA from DBA/1 between D1Mit55 and D1MitD1Mit209 inclusive. This region includes almost the entire DNA that was mapped within the QTL. Strain BALB.D1-2 contains genetic DNA from D1Mit359 through the end of the chromosome. Most of the DNA within this region is at the extreme distal end of the QTL. Thus, most of the transferred genomic region in BALB.D1-2 is not located within the QTL region identified in our initial mapping. The phenotype of BALB.D1-1^−/−^ was determined by observation of 18 mice from this strain over a period of 100 days (Figure 2). This congenic strain has significantly delayed onset of disease and reduced severity. (P<0.05 for incidence and <0.01 for severity) as compared to the parental BALB/c^−/−^ strain. The mean day of onset was delayed from day 60 to day 77. By 100 days of age, the congenic strain had an incidence of disease that was comparable to the parental strain i.e. 94% compared to 100%. However the severity was still reduced at 100 days in the congenic strain as compared to the parental. Shown in Figure 3 shows the histologic examination of a hind limb from a BALB/c^−/−^ compared to a BALB.D1-1^−/−^ mouse at 7 weeks of age. Both mice have arthritis but the severity of the BALB/c^−/−^ is much greater than that of the congenic.

**Figure 4:**
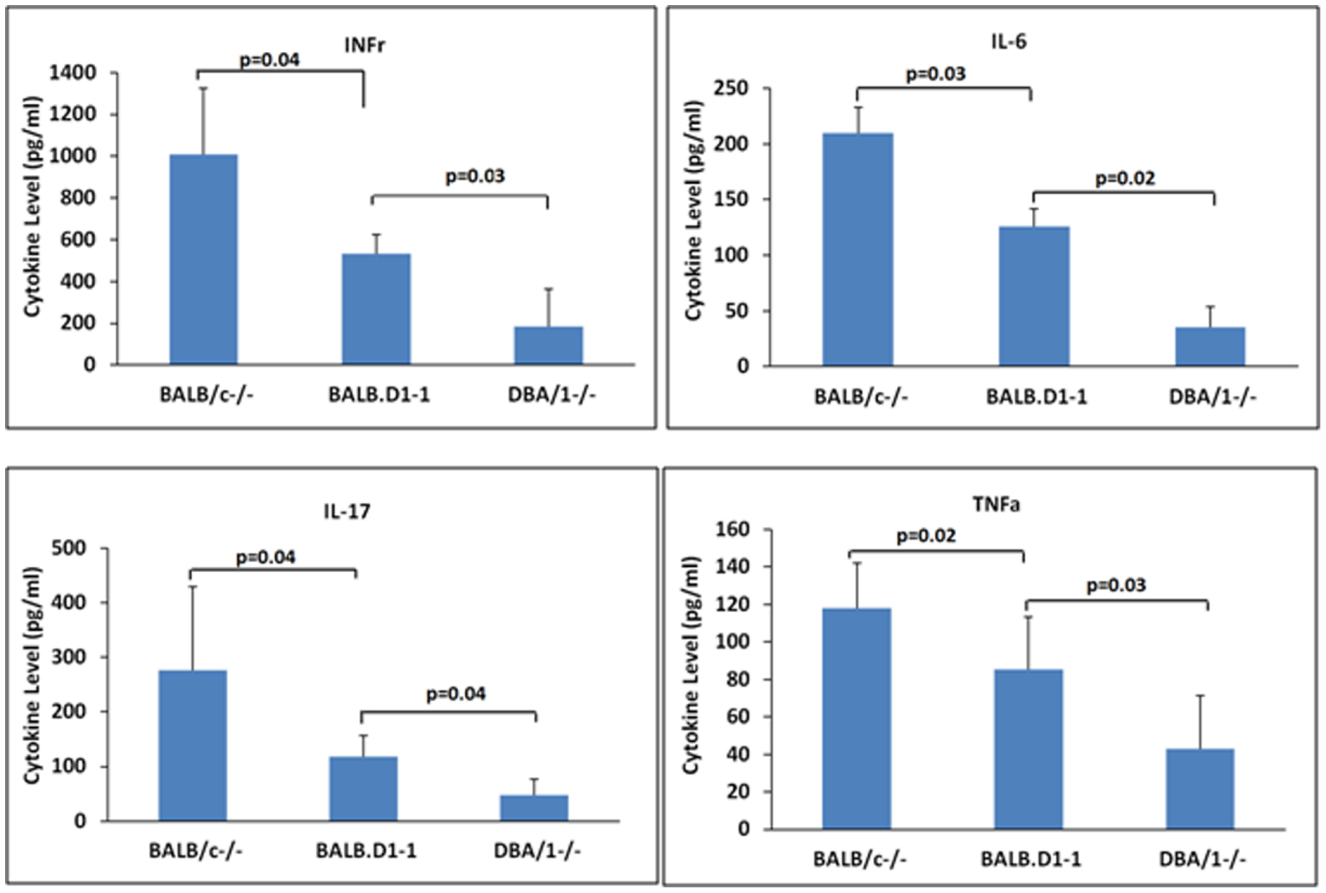
Production of cytokines by spleen cells from 4 month old mice. Cells were harvested from individual mice of each strain and cultured with CD3CD28 stimulation beads. Supernatant fluids were harvested after 48 hours of culture and assayed for the cytokines noted. Each bar represents the mean and standard deviation of 3 biologic replicates. Differences between goups was calculated by Student’s T test.

Although the etiology of the spontaneous arthritis has not yet been fully elucidated, it is known that disease is dependent on TNFα and IL-17 [14], [15]. We therefore analyzed the response of splenocytes from each strain to determine if there was differential regulation of these cytokines (Figure 4). For each of the cytokines studied i.e. TNFα, IL-17, IL-6 and IFNγ there was up regulation in the susceptible BALB/c as compared to DBA/1. The congenic strain showed intermediate levels. These data suggest that the QTL controls a generalized regulator of the immune response and not a specific regulator of IL-17 or TNFα.

Congenic strain BALB.D1-2^−/−^ contains a smaller piece of genomic region from DBA/1 than that in BALB.D1-1^−/−^. The region covers the distal region of the chromosome extending from at least D1MitD1Mit359 to the end of the chromosome. However, the phenotype of BALB.D1-2^−/−^ is similar to that of BALB.D1-1^−/−^. The phenotype of BALB.D1-2^−/−^ was established by observation of 15 mice. A control group consisted of 18 mice of the BALB/c^−/−^ strain (Figure 2). This strain also had significantly reduced severity and delayed onset of arthritis (P value <0.01 and <0.05 1for severity and incidence, respectively). The mean day of onset was day 68 for the congenic compared to 50 for the parental strain. Similar to BALB.D1-1^−/−^ by 100 days of age the congenic strain had an incidence disease of 87% compared to 100% for the parental controls. These data seem to confirm significant protection from spontaneous arthritis is conferred by the DNA from DBA/1 mice. The protection is only partial since the incidence of spontaneous arthritis ultimately approaches 100% in each of the strains tested. Interestingly, we did not find differential expression of *Il-1*, *Il-6* and *TNFα* between congenic strains and BALB/c^−/−^ although spleen cells were hyper responsive in BALB/c as compared to DBA/1 and were intermediate for the congenic strains as noted above.

### Genomic Region of QTL

Because of the similarity of protection from spontaneous arthritis in both the BALB.D1-1^−/−^ and BALB.D1-2^−/−^strains we assume that the DBA/1 DNA that is common between the two congenic strains contains the genetic factor/s that are responsible for the prevention of disease. By comparing the genetic markers in these two congenic strains, the region of interest can be reduced to a final common transferred region (Figure 1). The minimum common transferred region is between D1Mit359 and D1Mit209. The maximum transferred region can be from D1Mit110 to the distal end of the chromosome. The other two possibilities are between D1Mit110 and D1Mit209 and between D1Mit359 and distal end of the chromosome. Genomic region between D1Mit110 and D1Mit209 coincides with the downstream tail of the peak region identified in the initial map [8]. Therefore, it is likely that the genomic region between D1Mit110 (or a position close to D1Mit110) and D1Mit359 contains the causal gene/s for the QTL of SAD., Although D1Mit110 has a BABL/c genotype in the second congenic strain this does not seem to affect the development of disease or disease severity. The genes on interest would appear to be located within the genomic region between 167758517 (D1Mit110) and 191493284 bp (D1Mit209). This region is sentenic to human chromosome 1 in two regions, 1∶165171104 bp-158516918 bp and 1∶1∶240177648 bp-1∶209788220 bp. An exception is the gene *Alyref2*, on mouse 1∶171503478–171504750, which is located on human chromosome 17, between 17∶79845713 bp-79849462 bp.

### Candidate Genes within the QTL

Within the region of interest, there are 320 genes including 274 known genes, 41 novel genes, and 5 putative genes. Searching using key words (in our material and methods), PGMapper revealed 115 candidate genes according PubMed reports based on Ensembl (NCBI m37) (Table S2). However, the candidate genes favored based on our initial mapping [6], (*Mr1, Pla2g4a, Fasl, Prg4*, and *Ptgs2*) are not included in those genes. Analysis of known single nucleotide polymorphisms (SNPs) with the region of interest established that 32 of the 115 candidate genes have at least 1 polymorphism between BALB/cj and DBA/1j (Table S2). By analyzing the correlation of expression of those genes with *Il1rn* and its related genes, *Il1r, Il1a*, and *Il1b*, we fund none of the 115 genes has a high correlation with *Il1rn* and its related genes (Table S3). However, we found that 6 genes have correlation R value higher than 0.40 to at least one of the *Il1rn* related gene. Those 6 genes are *Fcgr3, Capn2, Dedd, Fcer1g, Tfb2m*, and *Ifi205*. Because we have previously established a list of 241 differentially expressed genes in comparison between BALB/c^−/−^ and DBA/1^−/−^ −/− mice, we compared the list of 115 candidates and the 241 differentially expressed genes. Our comparison indicated that 4 candidate genes are among the differentially expressed genes. Those 4 genes are *Fcer1g, Fcgr3, Ifi202b*, and *Kmo*. Interestingly, *Fcer1g, Fcgr3* are both show a correlation with gene expression of *Il1rn* as well as differential expression between spontaneous arthritis mice and healthy BALB/c mice. However, SNP data indicated that there are no known polymorphic SNPs between BALB/c and DBA/1 within those two genes and 2 kbp up and down stream.

## Discussion and Conclusion

Our congenic breeding was successful in identifying a QTL associated with the development of spontaneous arthritis. When a fragment of DNA from the DBA/1 strain was introduced onto a BALB/c background, arthritis was delayed in onset and was less severe. The congenic strains provide a unique tool for evaluating specific genetic factor/s that regulates the spontaneous onset of spontaneous arthritis. The genetic mapping of QTL using a F2 population, especially with a relatively small population, usually identifies approximate locations of genetic loci for a complex trait. Using the congenic strains that we developed, the genomic region of the originally identified QTL has been redefined into a region that is downstream from the peak region of our original mapping. In our previous study, in our F2 mapping we used 137 microsatellite markers with initially 191 F2 and then 561 F2 mice. Within the 561 F2 population, there is sex ratio of 1∶2 between male and female. This data emphasizes the importance of confirmation of QTL regions using additional breeding techniques including the development of congenic strains or other approaches.

Understanding the molecular mechanisms underlying the phenotype of the congenic strains has two potentially profound consequences. First, it may enable us to identify novel pathways that contribute to the development of inflammatory arthritis. It has been recognized that IL-1 signaling is a key component of many forms of human inflammatory arthritis, in the development of joint erosions, and in development of osteoporosis. Our data support the possibility that erosions are at least delayed in the congenic strains. They have not as yet been analyzed for bone density and we do not have QTL mapping of bone density in BALB/c^−/−^ X DBA/1^−/−^ mice. Second, because spontaneous arthritis develops independently of TNF, identification of novel signaling pathways may help to explain why some patients fail to respond to TNF inhibitors, Our analysis indicated that the QTL did not result changes in *Il1, Il6* and *Tnfα* or *Th17* and *Il17* based on expression levels in the splenocytes used for analysis. When splenocytes were isolated, cultured, stimulated by anti-CD3CD28 beads and analyzed for cytokine expression by quantitation of protein levels, it was evident that there was potential for different levels of expression based on the genotype. This data suggests that these cytokines may be coordinately regulated by genes within the QTL but further work will be needed to determine the specific pathways involved. Study of the molecular basis of this QTL may identify a complementary approach to what is the most widely implemented biologic therapy for inflammatory arthritis in humans. Although we have not yet identified the causal gene/s within the QTL, those genes with polymorphisms and differential expression levels between BALB/c and DBA/1 deserve detailed examination in the future. The mouse model in this study has important implications for understanding rheumatoid arthritis in humans and potentially other human diseases. We anticipate that the candidate gene/s in the QTL that regulate the spontaneous arthritis in the IL-1RN^−/−^ knockout mice may also be involved in human RA. 1) The effects of treatment of several inflammatory disorders with anakinra have been reported. Both positive and negative effects have been described [16]–[18]. Detailed study on the molecular function of *Il1rn* and its interaction with other genes or genetic factors is essential for development therapeutic application using *Il1rn*. 2) The genetic factors that interact with *Il1rn* may be an ideal target for the development therapeutic application.

We analyzed the common transferred region 2.4 Mbp (between 167758517 and 191493284 bp) (Figure 1) based in the overlapping markers of two congenics. This should be considered as the maximum size of the QTL. The actual size of the QTL could be between D1Mit 359 (located between 177285202-177285317) and D1Mit209, or between D1Mit110 and D1Mit426 (180411709–180411792) with the size of approximately 1.5 Mbp and 1.1 Mbp, respectively. Further break down within the current transferred region in the congenic strains will greatly reduce the number of the candidate genes for this QTL.

## Supporting Information

Figure S1 **Procedure of congenic breeding to show how the DBA/1 segment in QTL region is transferred into Babl/c genomic background.**
(PPTX)

Table S1 **Molecular markers used in the congenic breeding.**
(DOC)

Table S2 **Candidate genes within QTL region.**
(DOC)

Table S3 **Correlation between candidate genes and IL-1ra and its partner genes.**
(DOC)
