# Supplementary material for: Congenic Mice Provide Evidence for a Genetic Locus That Modulates Spontaneous Arthritis Caused by Deficiency of IL-1RA
Source: PLoS One. 2013 Jun 28;8(6):e68158. doi: 10.1371/journal.pone.0068158 (PMC3695999; doi:10.1371/journal.pone.0068158)
Supplement: Table S1 — Molecular markers used in the congenic breeding. (DOC) [file pone.0068158.s002.doc]

Supplementary Table S1. Molecular markers used in the congenic breeding

| **Chr #** | **Length of chr**  **(cM)** | **Microsatellite markers** |
| --- | --- | --- |
| 1 | 127 | D1Mit118, D1Mit67, D1Mit302, D1Mit156, D1Mit260, D1Mit445, D1Mit265, D1Mit425, D1Mit55, D1Mit506, D1Mit400, D1Mit110  D1Mit354, D1Mit403, D1Mit359, D1Mit426, D1Mit209 |
| 2 | 114 | D2Mit61, D2Mit305, D2Mit166, D2Mit305, D2Mit50, D2Mit230 |
| 3 | 95 | D3Mit268, D3Mit25, D3Mit199, D3Mit158, D3Mit200, D3Mit323 |
| 4 | 84 | D4Mit236, D4Mit142, D4Mit175, D4Mit233 |
| 5 | 92 | D5Mit354, D5Mit134, D5Mit177 |
| 6 | 75 | D6Mit291, D6Mit201, D6Mit223, D6Mit355, D6Mit67 |
| 7 | 74 | D7Mit77, D7Mit321, D7Mit101, D7Mit362, D7Mit83 |
| 8 | 82 | D8Mit357, D8Mit150, D8Mit272, D8Mit56, D8Mit289 |
| 9 | 79 | D9Mit297, D9Mit24, D9Mit19 |
| 10 | 77 | D10Mit3, D10Mit261, D10Mit209, D10Mit66, D10Mit231 |
| 11 | 80 | D11Mit151, D11Mit199, D11Mit151, D11Mit199 |
| 12 | 66 | D12Mit221, D12Mit158, D12Mit167 |
| 13 | 80 | D13Mit209, D13Mit235, D13MitMit60, D13Mit117, D13Mit26, D13Mit88, D13Mit75, D13Mit76 |
| 14 | 69 | D14Mit107, D14Mit149, D14Mit37, D14Mit30, D14Mit94 |
| 15 | 81 | D15Mit227, D15Mit161, D15Mit13, D15Mit26 |
| 16 | 72 | D16Mit57, D16Mit152 |
| 17 | 73 | D17Mit89, D17Mit122, D17Mit80 |
| 18 | 60 | D18Mit119, D18Mit152, D18Mit7 |
| 19 | 57 | D19Mit55, D19Mit88 |
