# Supplementary material for: Congenic Mice Provide Evidence for a Genetic Locus That Modulates Spontaneous Arthritis Caused by Deficiency of IL-1RA
Source: PLoS One. 2013 Jun 28;8(6):e68158. doi: 10.1371/journal.pone.0068158 (PMC3695999; doi:10.1371/journal.pone.0068158)
Supplement: Table S2 — Candidate genes within QTL region. (DOC) [file pone.0068158.s003.doc]

Supplementary Table S2. Candidate genes within QTL region.

| **Ensembl Accession** | **Description** | **Symbol** | **PubMed Hits** | **SNP number** | **SNP Function Class** | **Differential Expression** |
| --- | --- | --- | --- | --- | --- | --- |
| ENSMUSG00000037942 | C-reactive protein, pentraxin-related [Source:MGI Symbol;Acc:MGI:88512] | Crp | 13028 | 0 | - | - |
| ENSMUSG00000026542 | serum amyloid P-component [Source:MGI Symbol;Acc:MGI:98229] | Apcs | 3427 | 0 | - | - |
| ENSMUSG00000079164 | toll-like receptor 5 [Source:MGI Symbol;Acc:MGI:1858171] | Tlr5 | 554 | 0 | - | - |
| ENSMUSG00000015355 | CD48 antigen [Source:MGI Symbol;Acc:MGI:88339] | Cd48 | 197 | 0 | - | - |
| ENSMUSG00000026496 | poly (ADP-ribose) polymerase family, member 1 [Source:MGI Symbol;Acc:MGI:1340806] | Parp1 | 142 | 0 | - | - |
| ENSMUSG00000026663 | activating transcription factor 6 [Source:MGI Symbol;Acc:MGI:1926157] | Atf6 | 136 | 0 | - | - |
| ENSMUSG00000037860 | absent in melanoma 2 [Source:MGI Symbol;Acc:MGI:2686159] | Aim2 | 87 | 0 | - | - |
| ENSMUSG00000004709 | CD244 natural killer cell receptor 2B4 [Source:MGI Symbol;Acc:MGI:109294] | Cd244 | 80 | 0 | - | - |
| ENSMUSG00000006403 | a disintegrin-like and metallopeptidase (reprolysin type) with thrombospondin type 1 motif, 4 [Source:MGI Symbol;Acc:MGI:1339949] | Adamts4 | 79 | 0 | - | - |
| ENSMUSG00000037872 | Duffy blood group, chemokine receptor [Source:MGI Symbol;Acc:MGI:1097689] | Darc | 75 | 0 | - | - |
| ENSMUSG00000039748 | exonuclease 1 [Source:MGI Symbol;Acc:MGI:1349427] | Exo1 | 73 | 0 | - | - |
| ENSMUSG00000052534 | pre B-cell leukemia transcription factor 1 [Source:MGI Symbol;Acc:MGI:97495] | Pbx1 | 71 | 0 | - | - |
| ENSMUSG00000026656 | Fc receptor, IgG, low affinity IIb [Source:MGI Symbol;Acc:MGI:95499] | Fcgr2b | 54 | 0 | - | - |
| ENSMUSG00000019699 | thymoma viral proto-oncogene 3 [Source:MGI Symbol;Acc:MGI:1345147] | Akt3 | 47 | 0 | - | - |
| ENSMUSG00000026674 | discoidin domain receptor family, member 2 [Source:MGI Symbol;Acc:MGI:1345277] | Ddr2 | 45 | 0 | - | - |
| ENSMUSG00000038147 | CD84 antigen [Source:MGI Symbol;Acc:MGI:1336885] | Cd84 | 39 | 0 | - | - |
| ENSMUSG00000004880 | lamin B receptor [Source:MGI Symbol;Acc:MGI:2138281] | Lbr | 37 | 0 | - | - |
| ENSMUSG00000038530 | regulator of G-protein signaling 4 [Source:MGI Symbol;Acc:MGI:108409] | Rgs4 | 34 | 0 | - | - |
| ENSMUSG00000026678 | regulator of G-protein signaling 5 [Source:MGI Symbol;Acc:MGI:1098434] | Rgs5 | 33 | rs13476230 | Coding-Synonymous | - |
| ENSMUSG00000039239 | transforming growth factor, beta 2 [Source:MGI Symbol;Acc:MGI:98726] | Tgfb2 | 31 | rs13476289 | [Tgfb2 : Intron](http://www.informatics.jax.org/marker/MGI:98726) | - |
| ENSMUSG00000038776 | epoxide hydrolase 1, microsomal [Source:MGI Symbol;Acc:MGI:95405] | Ephx1 | 30 | 0 | - | - |
| ENSMUSG00000026536 | myeloid cell nuclear differentiation antigen [Source:MGI Symbol;Acc:MGI:3041120] | Mnda | 25 | 0 | - | - |
| ENSMUSG00000026641 | upstream transcription factor 1 [Source:MGI Symbol;Acc:MGI:99542] | Usf1 | 25 | 0 | - | - |
| ENSMUSG00000039377 | H2.0-like homeobox [Source:MGI Symbol;Acc:MGI:96109] | Hlx | 24 | 0 | - | - |
| ENSMUSG00000015316 | signaling lymphocytic activation molecule family member 1 [Source:MGI Symbol;Acc:MGI:1351314] | Slamf1 | 22 | 0 | - | - |
| ENSMUSG00000026553 | coatomer protein complex subunit alpha [Source:MGI Symbol;Acc:MGI:1334462] | Copa | 21 | 0 | - | - |
| ENSMUSG00000058076 | succinate dehydrogenase complex, subunit C, integral membrane protein [Source:MGI Symbol;Acc:MGI:1913302] | Sdhc | 20 | 0 | - | - |
| ENSMUSG00000004707 | lymphocyte antigen 9 [Source:MGI Symbol;Acc:MGI:96885] | Ly9 | 18 | 0 | - | - |
| ENSMUSG00000039783 | kynurenine 3-monooxygenase (kynurenine 3-hydroxylase) [Source:MGI Symbol;Acc:MGI:2138151] | Kmo | 15 | rs6299624 | Intron | Y |
| ENSMUSG00000005677 | nuclear receptor subfamily 1, group I, member 3 [Source:MGI Symbol;Acc:MGI:1346307] | Nr1i3 | 15 | 0 | - | - |
| ENSMUSG00000056569 | myelin protein zero [Source:MGI Symbol;Acc:MGI:103177] | Mpz | 15 | 0 | - | - |
| ENSMUSG00000010609 | presenilin 2 [Source:MGI Symbol;Acc:MGI:109284] | Psen2 | 13 | 0 | - | - |
| ENSMUSG00000059498 | Fc receptor, IgG, low affinity III [Source:MGI Symbol;Acc:MGI:95500] | Fcgr3 | 13 | 0 |  | Y |
| ENSMUSG00000026526 | fumarate hydratase 1 [Source:MGI Symbol;Acc:MGI:95530] | Fh1 | 12 | rs13476256 | Coding-Synonymous | - |
| ENSMUSG00000015314 | SLAM family member 6 [Source:MGI Symbol;Acc:MGI:1353620] | Slamf6 | 12 | rs13476238 | Intron | - |
| ENSMUSG00000038235 | F11 receptor [Source:MGI Symbol;Acc:MGI:1321398] | F11r | 12 |  | - | - |
| ENSMUSG00000026509 | calpain 2 [Source:MGI Symbol;Acc:MGI:88264] | Capn2 | 11 | rs8280764 | Intron | - |
| ENSMUSG00000039997 | interferon activated gene 203 [Source:MGI Symbol;Acc:MGI:96428] | Ifi203 | 11 | rs13476244 | within coordinates of | - |
| ENSMUSG00000005339 | Fc receptor, IgE, high affinity I, alpha polypeptide [Source:MGI Symbol;Acc:MGI:95494] | Fcer1a | 11 | rs8242509 | Intron | - |
| ENSMUSG00000026688 | microsomal glutathione S-transferase 3 [Source:MGI Symbol;Acc:MGI:1913697] | Mgst3 | 11 | rs13476222 | Intron | - |
| ENSMUSG00000073489 | interferon activated gene 204 [Source:MGI Symbol;Acc:MGI:96429] | Ifi204 | 10 | 0 | - | - |
| ENSMUSG00000013973 | death effector domain-containing [Source:MGI Symbol;Acc:MGI:1333874] | Dedd | 10 | 0 | - | - |
| ENSMUSG00000026686 | LIM homeobox transcription factor 1 alpha [Source:MGI Symbol;Acc:MGI:1888519] | Lmx1a | 10 | rs13476223 | Intron | - |
| ENSMUSG00000026615 | glutamyl-prolyl-tRNA synthetase [Source:MGI Symbol;Acc:MGI:97838] | Eprs | 9 | 0 | - | - |
| ENSMUSG00000015961 | adenylosuccinate synthetase, non muscle [Source:MGI Symbol;Acc:MGI:87948] | Adss | 9 | 0 | - | - |
| ENSMUSG00000026527 | regulator of G protein signaling 7 [Source:MGI Symbol;Acc:MGI:1346089] | Rgs7 | 9 | 0 | - | - |
| ENSMUSG00000005681 | apolipoprotein A-II [Source:MGI Symbol;Acc:MGI:88050] | Apoa2 | 9 | rs3022859 | Coding-Synonymous | - |
| ENSMUSG00000038179 | SLAM family member 7 [Source:MGI Symbol;Acc:MGI:1922595] | Slamf7 | 8 | 0 | - | - |
| ENSMUSG00000058715 | Fc receptor, IgE, high affinity I, gamma polypeptide [Source:MGI Symbol;Acc:MGI:95496] | Fcer1g | 8 | 0 | - | Y |
| ENSMUSG00000026535 | interferon activated gene 202B [Source:MGI Symbol;Acc:MGI:1347083] | Ifi202b | 7 | 0 | - | Y |
| ENSMUSG00000007097 | ATPase, Na+/K+ transporting, alpha 2 polypeptide [Source:MGI Symbol;Acc:MGI:88106] | Atp1a2 | 7 | 0 | - | - |
| ENSMUSG00000013997 | nitrilase 1 [Source:MGI Symbol;Acc:MGI:1350916] | Nit1 | 7 | rs4222839 | [Nit1 : Intron; Pfdn2 : Locus-Region (upstream)](http://www.informatics.jax.org/marker/MGI:1350916) | - |
| ENSMUSG00000015843 | retinoid X receptor gamma [Source:MGI Symbol;Acc:MGI:98216] | Rxrg | 7 | 0 | - | - |
| ENSMUSG00000039384 | dual specificity phosphatase 10 [Source:MGI Symbol;Acc:MGI:1927070] | Dusp10 | 6 | rs13476280 | Intron | - |
| ENSMUSG00000026516 | nuclear VCP-like [Source:MGI Symbol;Acc:MGI:1914709] | Nvl | 6 |  |  | - |
| ENSMUSG00000007122 | calsequestrin 1 [Source:MGI Symbol;Acc:MGI:1309468] | Casq1 | 6 | rs13476239 | 1893 bp upstream of | - |
| ENSMUSG00000062729 | protoporphyrinogen oxidase [Source:MGI Symbol;Acc:MGI:104968] | Ppox | 6 | rs4222835 | B4galt3 : Coding-Synonymous; Ppox : Locus-Region | - |
| ENSMUSG00000040629 | maelstrom homolog (Drosophila) [Source:MGI Symbol;Acc:MGI:2138453] | Mael | 6 | 0 |  | - |
| ENSMUSG00000055067 | SET and MYND domain containing 3 [Source:MGI Symbol;Acc:MGI:1916976] | Smyd3 | 5 | rs13459055 | Coding-NonSynonymous | - |
|  |  |  |  | rs6364466 | Intron | - |
|  |  |  |  | rs13476264 | Intron | - |
| ENSMUSG00000003464 | peroxisomal biogenesis factor 19 [Source:MGI Symbol;Acc:MGI:1334458] | Pex19 | 5 | 0 | - | - |
| ENSMUSG00000026556 | vang-like 2 (van gogh, Drosophila) [Source:MGI Symbol;Acc:MGI:2135272] | Vangl2 | 5 | 0 | - | - |
| ENSMUSG00000006411 | poliovirus receptor-related 4 [Source:MGI Symbol;Acc:MGI:1918990] | Pvrl4 | 5 | 0 | - | - |
| ENSMUSG00000026620 | MAP/microtubule affinity-regulating kinase 1 [Source:MGI Symbol;Acc:MGI:2664902] | Mark1 | 4 | rs13476283 | Coding-NonSynonymous | - |
| ENSMUSG00000066652 | left-right determination factor 2 [Source:MGI Symbol;Acc:MGI:2443573] | Lefty2 | 4 | 0 | - | - |
| ENSMUSG00000003458 | nicastrin [Source:MGI Symbol;Acc:MGI:1891700] | Ncstn | 4 | 0 | - | - |
| ENSMUSG00000051251 | nescient helix loop helix 1 [Source:MGI Symbol;Acc:MGI:98481] | Nhlh1 | 4 | 0 | - | - |
| ENSMUSG00000026565 | POU domain, class 2, transcription factor 1 [Source:MGI Symbol;Acc:MGI:101898] | Pou2f1 | 4 | rs6363233 | Intron | - |
| ENSMUSG00000026605 | centromere protein F [Source:MGI Symbol;Acc:MGI:1313302] | Cenpf | 3 | 0 | - | - |
| ENSMUSG00000037624 | potassium channel, subfamily K, member 2 [Source:MGI Symbol;Acc:MGI:109366] | Kcnk2 | 3 | rs13476296 | Intron | - |
| ENSMUSG00000022995 | enabled homolog (Drosophila) [Source:MGI Symbol;Acc:MGI:108360] | Enah | 3 | 0 | - | - |
| ENSMUSG00000026497 | Mix1 homeobox-like 1 (Xenopus laevis) [Source:MGI Symbol;Acc:MGI:1351322] | Mixl1 | 3 | 0 | - | - |
| ENSMUSG00000038855 | inositol 1,4,5-trisphosphate 3-kinase B [Source:MGI Symbol;Acc:MGI:109235] | Itpkb | 3 | 0 | - | - |
| ENSMUSG00000026492 | transcription factor B2, mitochondrial [Source:MGI Symbol;Acc:MGI:107937] | Tfb2m | 3 | 0 | - | - |
| ENSMUSG00000039630 | heterogeneous nuclear ribonucleoprotein U [Source:MGI Symbol;Acc:MGI:1858195] | Hnrnpu | 3 | 0 | - | - |
| ENSMUSG00000054203 | interferon activated gene 205 [Source:MGI Symbol;Acc:MGI:101847] | Ifi205 | 3 | 0 | - | - |
| ENSMUSG00000070504 | Fc receptor-like 6 [Source:MGI Symbol;Acc:MGI:3618339] | Fcrl6 | 3 | 0 | - | - |
| ENSMUSG00000050229 | phosphatidylinositol glycan anchor biosynthesis, class M [Source:MGI Symbol;Acc:MGI:1914806] | Pigm | 3 | 0 | - | - |
| ENSMUSG00000062963 | ubiquitin-fold modifier conjugating enzyme 1 [Source:MGI Symbol;Acc:MGI:1913405] | Ufc1 | 3 | 0 | - | - |
| ENSMUSG00000013593 | NADH dehydrogenase (ubiquinone) Fe-S protein 2 [Source:MGI Symbol;Acc:MGI:2385112] | Ndufs2 | 3 | rs8245216 | Coding-Synonymous | - |
| ENSMUSG00000038421 | Fc receptor-like A [Source:MGI Symbol;Acc:MGI:2138647] | Fcrla | 3 |  | - | - |
| ENSMUSG00000038473 | nitric oxide synthase 1 (neuronal) adaptor protein [Source:MGI Symbol;Acc:MGI:1917979] | Nos1ap | 3 | rs13476231 | 1700015E13Rik : Coding-Synonymous; Nos1ap : within coordinates of | - |
|  |  |  |  | rs13476232 | Intron | - |
| ENSMUSG00000026558 | uridine-cytidine kinase 2 [Source:MGI Symbol;Acc:MGI:1931744] | Uck2 | 3 | 0 | - | - |
| ENSMUSG00000053664 | uridine-cytidine kinase 2 [Source:MGI Symbol;Acc:MGI:1931744] | Uck2 | 3 | 0 | - | - |
| ENSMUSG00000026610 | estrogen-related receptor gamma [Source:MGI Symbol;Acc:MGI:1347056] | Esrrg | 2 | rs13476291 | within coordinates of | - |
|  |  |  |  | rs13476292 | within coordinates of | - |
| ENSMUSG00000026511 | signal recognition particle 9 [Source:MGI Symbol;Acc:MGI:1350930] | Srp9 | 2 | 0 | - | - |
| ENSMUSG00000038793 | left right determination factor 1 [Source:MGI Symbol;Acc:MGI:107405] | Lefty1 | 2 | 0 | - | - |
| ENSMUSG00000058729 | lin-9 homolog (C. elegans) [Source:MGI Symbol;Acc:MGI:1919818] | Lin9 | 2 | 0 | - | - |
| ENSMUSG00000078185 | choroideremia-like [Source:MGI Symbol;Acc:MGI:101913] | Chml | 2 | 0 | - | - |
| ENSMUSG00000026525 | opsin 3 [Source:MGI Symbol;Acc:MGI:1338022] | Opn3 | 2 | 0 | - | - |
| ENSMUSG00000050069 | gremlin 2 homolog, cysteine knot superfamily (Xenopus laevis) [Source:MGI Symbol;Acc:MGI:1344367] | Grem2 | 2 | 0 | - | - |
| ENSMUSG00000053318 | SLAM family member 8 [Source:MGI Symbol;Acc:MGI:1921998] | Slamf8 | 2 | 0 | - | - |
| ENSMUSG00000026547 | transgelin 2 [Source:MGI Symbol;Acc:MGI:1312985] | Tagln2 | 2 | 0 | - | - |
| ENSMUSG00000044708 | potassium inwardly-rectifying channel, subfamily J, member 10 [Source:MGI Symbol;Acc:MGI:1194504] | Kcnj10 | 2 | 0 | - | - |
| ENSMUSG00000038209 | intelectin 1 (galactofuranose binding) [Source:MGI Symbol;Acc:MGI:1333831] | Itln1 | 2 | rs13476235 | Coding-Synonymous | - |
| ENSMUSG00000026659 | dual specificity phosphatase 12 [Source:MGI Symbol;Acc:MGI:1890614] | Dusp12 | 2 | rs13476246 | Coding-Synonymous | - |
| ENSMUSG00000026564 | dual specificity phosphatase 27 (putative) [Source:MGI Symbol;Acc:MGI:2685055] | Dusp27 | 2 | 0 | - | - |
| ENSMUSG00000026510 | transformation related protein 53 binding protein 2 [Source:MGI Symbol;Acc:MGI:2138319] | Trp53bp2 | 1 | 0 | - | - |
| ENSMUSG00000038633 | degenerative spermatocyte homolog 1 (Drosophila) [Source:MGI Symbol;Acc:MGI:1097711] | Degs1 | 1 | rs13476274 | Coding-Synonymous | - |
| ENSMUSG00000038733 | WD repeat domain 26 [Source:MGI Symbol;Acc:MGI:1923825] | Wdr26 | 1 | 0 | - | - |
| ENSMUSG00000062169 | cornichon homolog 4 (Drosophila) [Source:MGI Symbol;Acc:MGI:1925828] | Cnih4 | 1 | 0 | - | - |
| ENSMUSG00000060743 | H3 histone, family 3A [Source:MGI Symbol;Acc:MGI:1097686] | H3f3a | 1 | 0 | - | - |
| ENSMUSG00000026499 | acyl-Coenzyme A binding domain containing 3 [Source:MGI Symbol;Acc:MGI:2181074] | Acbd3 | 1 | 0 | - | - |
| ENSMUSG00000038949 | consortin, connexin sorting protein [Source:MGI Symbol;Acc:MGI:2445141] | Cnst | 1 | rs13476265 | Coding-Synonymous | - |
| ENSMUSG00000026494 | kinesin family member 26B [Source:MGI Symbol;Acc:MGI:2447076] | Kif26b | 1 | rs13476263 | within coordinates of | - |
| ENSMUSG00000026504 | serologically defined colon cancer antigen 8 [Source:MGI Symbol;Acc:MGI:1924066] | Sdccag8 | 1 | rs13476258 | Intron | - |
| ENSMUSG00000090272 | myeloid nuclear differentiation antigen like [Source:MGI Symbol;Acc:MGI:3780953] | Mndal | 1 | rs13476244 * | within coordinates of | - |
| ENSMUSG00000005338 | cell adhesion molecule 3 [Source:MGI Symbol;Acc:MGI:2137858] | Cadm3 | 1 | rs13476242 | Intron | - |
| ENSMUSG00000026544 | dual specificity phosphatase 23 [Source:MGI Symbol;Acc:MGI:1915690] | Dusp23 | 1 | 0 | - | - |
| ENSMUSG00000038034 | immunoglobulin superfamily, member 8 [Source:MGI Symbol;Acc:MGI:2154090] | Igsf8 | 1 | 0 | - | - |
| ENSMUSG00000007107 | ATPase, Na+/K+ transporting, alpha 4 polypeptide [Source:MGI Symbol;Acc:MGI:1351335] | Atp1a4 | 1 | rs13476239 | 1752 bp downstream of | - |
| ENSMUSG00000053483 | ubiquitin specific peptidase 21 [Source:MGI Symbol;Acc:MGI:1353665] | Usp21 | 1 | 0 | - | - |
| ENSMUSG00000026675 | hydroxysteroid (17-beta) dehydrogenase 7 [Source:MGI Symbol;Acc:MGI:1330808] | Hsd17b7 | 1 | 0 | - | - |
| ENSMUSG00000026683 | NUF2, NDC80 kinetochore complex component, homolog (S. cerevisiae) [Source:MGI Symbol;Acc:MGI:1914227] | Nuf2 | 1 | rs13459163 | Intron | - |
| ENSMUSG00000026687 | aldehyde dehydrogenase 9, subfamily A1 [Source:MGI Symbol;Acc:MGI:1861622] | Aldh9a1 | 1 | 0 | - | - |
| ENSMUSG00000052428 | transmembrane and coiled-coil domains 1 [Source:MGI Symbol;Acc:MGI:1921173] | Tmco1 | 1 | 0 | - | - |
| ENSMUSG00000060568 | family with sequence similarity 78, member B [Source:MGI Symbol;Acc:MGI:2443050] | Fam78b | 1 | 0 | - | - |
