# Supplementary material for: Congenic Mice Provide Evidence for a Genetic Locus That Modulates Spontaneous Arthritis Caused by Deficiency of IL-1RA
Source: PLoS One. 2013 Jun 28;8(6):e68158. doi: 10.1371/journal.pone.0068158 (PMC3695999; doi:10.1371/journal.pone.0068158)
Supplement: Table S3 — Correlation between candidate genes and IL-1ra and its partner genes. (DOC) [file pone.0068158.s004.doc]

Supplementary Table S3. Correlation between candidate genes and IL-1ra and its partner genes.

| **Symbol** | **Spleen, Il1a** | **Spleen, Il1b** | **Spleen, Il1r1** | **Spleen, Il1rn** |
| --- | --- | --- | --- | --- |
| Crp | -0.114 | -0.011 | 0.02 | 0.128 |
| Apcs | -0.129 | 0.027 | -0.062 | 0.162 |
| Tlr5 |  |  |  |  |
| Cd48 | 0.34 | 0.198 | 0.24 | 0.101 |
| Parp1 | 0.131 | -0.118 | 0.218 | -0.028 |
| Atf6 | 0.197 | 0.204 | 0.344 | 0.045 |
| Aim2 | 0.399 | 0.267 | 0.365 | 0.131 |
| Cd244 | 0.265 | 0.393 | 0.142 | 0.114 |
| Adamts4 | -0.107 | -0.018 | -0.132 | 0.022 |
| Darc | -0.203 | -0.24 | -0.242 | -0.11 |
| Exo1 | -0.14 | -0.25 | -0.271 | -0.095 |
| Pbx1 | 0.26 | 0.319 | 0.412 | 0.238 |
| Fcgr2b | 0.308 | 0.163 | 0.306 | 0.046 |
| Akt3 | 0.158 | 0.193 | 0.216 | -0.118 |
| Ddr2 | 0.101 | 0 | 0.303 | -0.083 |
| Cd84 | 0.294 | 0.308 | 0.133 | 0.113 |
| Lbr | -0.082 | -0.264 | -0.066 | -0.114 |
| Rgs4 | 0.173 | 0.085 | 0.302 | 0.067 |
| Rgs5 | 0.085 | -0.007 | 0.219 | 0.006 |
| Rgs5 | 0.062 | 0.01 | 0.207 | -0.013 |
| Tgfb2 | -0.027 | 0.004 | 0.106 | 0.182 |
| Tgfb2 | -0.299 | -0.062 | -0.239 | 0.106 |
| Ephx1 | 0.328 | -0.047 | 0.396 | 0.122 |
| Mnda | X | X | X | X |
| Usf1 | 0.206 | 0.175 | 0.081 | 0.14 |
| Hlx | 0.314 | 0.325 | 0.341 | 0.391 |
| Slamf1 | 0.324 | 0.123 | 0.251 | 0.207 |
| Copa | 0.317 | 0.029 | 0.437 | 0.129 |
| Copa | 0.17 | 0.021 | 0.028 | 0.011 |
| Sdhc | -0.059 | -0.002 | 0.067 | 0.133 |
| Ly9 | 0.369 | 0.09 | 0.278 | 0.099 |
| Kmo | 0.263 | 0.178 | 0.293 | 0.086 |
| Nr1i3 | -0.298 | -0.118 | -0.184 | 0.112 |
| Mpz | -0.205 | -0.214 | -0.252 | 0.136 |
| Psen2 | 0.323 | 0.066 | 0.383 | 0.142 |
| Fcgr3 | 0.332 | 0.244 | 0.096 | 0.248 |
| Fcgr3 | 0.456 | 0.401 | 0.277 | 0.464 |
| Fh1 | 0.197 | 0.016 | -0.024 | 0.059 |
| Slamf6 | 0.16 | 0.101 | 0.329 | 0.014 |
| F11r | 0.339 | 0.264 | 0.171 | 0.325 |
| Capn2 | 0.393 | 0.24 | 0.451 | 0.237 |
| Ifi203 | 0.23 | 0.072 | 0.055 | 0.003 |
| Fcer1a | 0.202 | -0.062 | 0.171 | 0.158 |
| Mgst3 | -0.217 | -0.173 | -0.321 | -0.152 |
| Ifi204 | 0.331 | 0.218 | -0.017 | 0.166 |
| Dedd | 0.404 | 0.258 | 0.327 | 0.015 |
| Lmx1a | -0.181 | 0.032 | -0.154 | 0.338 |
| Eprs | -0.018 | -0.176 | 0.004 | -0.202 |
| Adss | 0.134 | -0.005 | 0.114 | 0.297 |
| Rgs7 | 0.184 | -0.071 | 0.291 | 0.153 |
| Apoa2 | 0.032 | 0.145 | -0.085 | 0.049 |
| Slamf7 | 0.304 | 0.126 | 0.302 | 0.149 |
| Fcer1g | 0.412 | 0.278 | 0.084 | 0.338 |
| Ifi202b | -0.117 | 0.025 | 0.014 | 0.116 |
| Atp1a2 | -0.08 | -0.18 | 0.15 | -0.028 |
| Nit1 | 0.369 | 0.138 | 0.267 | 0.043 |
| Rxrg | 0.213 | -0.128 | 0.338 | 0.051 |
| Dusp10 | 0.227 | -0.057 | 0.171 | 0.07 |
| Nvl | 0.092 | -0.097 | 0.132 | -0.164 |
| Casq1 | -0.207 | 0.005 | 0.008 | 0.034 |
| Ppox | -0.223 | -0.284 | -0.308 | -0.173 |
| Mael | 0.128 | -0.024 | 0.06 | 0.072 |
| Smyd3 | 0.289 | 0.175 | 0.394 | -0.076 |
|  |  |  |  |  |
|  |  |  |  |  |
| Pex19 | 0.264 | -0.01 | 0.299 | 0.062 |
| Vangl2 | 0.023 | 0.023 | -0.136 | 0.181 |
| Pvrl4 | -0.043 | -0.043 | -0.08 | 0.22 |
| Mark1 | 0.109 | 0.057 | 0.233 | 0.001 |
| Lefty2 | -0.174 | -0.157 | -0.284 | 0.105 |
| Ncstn | 0.211 | -0.057 | 0.253 | 0.127 |
| Ncstn | 0.07 | -0.016 | -0.033 | 0.045 |
| Nhlh1 | -0.13 | -0.114 | -0.012 | -0.017 |
| Pou2f1 | 0.056 | -0.002 | 0.163 | -0.169 |
| Cenpf | -0.168 | -0.22 | -0.201 | -0.092 |
| Kcnk2 | -0.228 | 0.01 | -0.347 | 0.017 |
| Enah | 0.017 | 0.074 | 0.094 | 0.16 |
| Mixl1 | -0.111 | 0.074 | -0.102 | 0.059 |
| Itpkb | 0.099 | 0.052 | 0.246 | -0.077 |
| Tfb2m | 0.388 | 0.031 | 0.417 | 0.071 |
| Tfb2m | 0.399 | 0.028 | 0.412 | 0.086 |
| Hnrnpu | -0.166 | -0.18 | -0.13 | -0.223 |
| Ifi205 | 0.374 | 0.224 | 0.497 | 0.145 |
| Fcrl6 | -0.177 | -0.05 | -0.076 | 0.069 |
| Pigm | 0.028 | -0.069 | 0.04 | -0.254 |
| Ufc1 | 0.153 | 0.129 | 0.077 | 0.199 |
| Ndufs2 | 0.031 | 0.039 | 0.067 | 0.165 |
| Fcrla | 0.321 | 0.02 | 0.319 | 0.187 |
| Nos1ap | -0.289 | -0.042 | -0.169 | -0.176 |
|  | -0.151 | -0.116 | -0.171 | 0.061 |
| Uck2 | -0.253 | -0.18 | -0.196 | -0.023 |
| Uck2 | -0.066 | -0.158 | -0.13 | 0.119 |
| Esrrg | -0.178 | -0.098 | -0.155 | 0.211 |
|  |  |  |  |  |
| Srp9 | 0.169 | -0.008 | 0.132 | 0.151 |
| Srp9 | -0.091 | 0.161 | -0.11 | 0.101 |
| Srp9 | -0.054 | 0.103 | -0.127 | 0.015 |
| Lefty1 | 0.015 | 0.025 | -0.135 | 0.142 |
| Lin9 | 0.135 | -0.092 | 0.121 | 0.032 |
| Chml | 0.183 | -0.004 | 0.119 | -0.183 |
| Opn3 | -0.113 | -0.222 | -0.088 | 0.028 |
| Grem2 | -0.176 | -0.099 | -0.01 | 0.076 |
| Slamf8 | 0.342 | 0.176 | 0.217 | 0.133 |
| Tagln2 | 0.326 | 0.153 | 0.107 | 0.253 |
| Kcnj10 | 0.185 | 0.028 | 0.146 | 0.048 |
| Itln1 | -0.048 | 0.056 | 0.034 | 0.02 |
| Dusp12 | 0.24 | 0.267 | 0.366 | -0.088 |
| Dusp12 | -0.325 | -0.194 | -0.274 | 0.097 |
| Dusp27 | -0.061 | -0.036 | -0.176 | 0.167 |
| Trp53bp2 | 0.08 | 0.064 | 0.26 | -0.022 |
| Degs1 | 0.263 | 0.281 | 0.346 | 0.245 |
| Wdr26 | -0.256 | -0.194 | -0.154 | -0.234 |
| Cnih4 | 0.304 | 0.146 | 0.275 | 0.229 |
| H3f3a | -0.14 | -0.094 | -0.358 | -0.165 |
|  | -0.159 | -0.102 | -0.351 | -0.237 |
|  | -0.14 | -0.071 | -0.335 | -0.214 |
| Acbd3 | 0.114 | -0.017 | 0.218 | -0.019 |
| Cnst | -0.045 | -0.049 | -0.086 | -0.075 |
| Kif26b | -0.287 | -0.224 | -0.312 | 0.135 |
|  | -0.152 | -0.008 | -0.175 | 0.191 |
| Sdccag8 | 0.156 | 0.183 | 0.197 | -0.16 |
| Mndal | X | X | X | X |
| Cadm3 | 0.174 | 0.138 | 0.135 | 0.194 |
| Dusp23 | -0.053 | -0.032 | -0.109 | 0.088 |
| Igsf8 | -0.238 | -0.182 | -0.128 | 0.01 |
| Atp1a4 | -0.065 | 0.053 | -0.028 | 0.162 |
| Usp21 | 0.251 | -0.089 | 0.147 | 0.103 |
| Hsd17b7 | 0.188 | -0.119 | 0.354 | 0.206 |
| Nuf2 | -0.037 | -0.188 | -0.158 | 0.006 |
| Aldh9a1 | 0.059 | -0.222 | 0.219 | 0.113 |
| Tmco1 | 0.124 | 0.213 | -0.093 | 0.119 |
| Fam78b | -0.077 | -0.081 | -0.035 | 0.11 |
